# Fabrication and Characterization of an Electrospun PHA/Graphene Silver Nanocomposite Scaffold for Antibacterial Applications

**DOI:** 10.3390/ma11091673

**Published:** 2018-09-10

**Authors:** Abdul Mukheem, Kasturi Muthoosamy, Sivakumar Manickam, Kumar Sudesh, Syed Shahabuddin, Rahman Saidur, Noor Akbar, Nanthini Sridewi

**Affiliations:** 1 Department of Maritime Science and Technology Faculty of Science and Defence Technology, National Defence University of Malaysia, Kuala Lumpur 57000, Malaysia; 2 Department of Chemical and Environmental Engineering, Faculty of Engineering, University of Nottingham Malaysia Campus, Semenyih 43500, Malaysia; Kasturi.Muthoosamy@nottingham.edu.my (K.M.); Sivakumar.Manickam@nottingham.edu.my (S.M.); 3 Applied Microbiology and Ecobiomaterial Research Laboratory, School of Biological Sciences, Universiti Sains Malaysia, Penang 11800, Malaysia; ksudesh@usm.my; 4 Research Centre for Nano-Materials and Energy Technology (RCNMET), School of Science and Technology, Sunway University, Subang Jaya 47500, Malaysia; saidur@sunway.edu.my; 5 School of Postgraduate Studies and Research, American University of Ras Al Khaimah, Ras Al Khaimah 31208, UAE; 6 Department of Biological Sciences, School of Science and Technology, Sunway University, Subang Jaya 47500, Malaysia; noormicrobiologist555@gmail.com

**Keywords:** graphene, silver nanoparticles, PHA, electrospun biomaterial, antibacterial

## Abstract

Many wounds are unresponsive to currently available treatment techniques and therefore there is an immense need to explore suitable materials, including biomaterials, which could be considered as the crucial factor to accelerate the healing cascade. In this study, we fabricated polyhydroxyalkanoate-based antibacterial mats via an electrospinning technique. One-pot green synthesized graphene-decorated silver nanoparticles (GAg) were incorporated into the fibres of poly-3 hydroxybutarate-co-12 mol.% hydroxyhexanoate (P3HB-co-12 mol.% HHx), a co-polymer of the polyhydroxyalkanoate (PHA) family which is highly biocompatible, biodegradable, and flexible in nature. The synthesized PHA/GAg biomaterial has been characterized by field emission scanning electron microscopy (FESEM), elemental mapping, thermogravimetric analysis (TGA), UV-visible spectroscopy (UV-vis), and Fourier transform infrared spectroscopy (FTIR). An in vitro antibacterial analysis was performed to investigate the efficacy of PHA/GAg against gram-positive *Staphylococcus aureus* (*S. aureus*) strain 12,600 ATCC and gram-negative *Escherichia coli* (*E. coli*) strain 8739 ATCC. The results indicated that the PHA/GAg demonstrated significant reduction of *S. aureus* and *E. coli* as compared to bare PHA or PHA- reduced graphene oxide (rGO) in 2 h of time. The *p* value (*p* < 0.05) was obtained by using a two-sample *t*-test distribution.

## 1. Introduction

The skin is one of the largest organs of the human body and has many functions. The key function of the skin is to protect the human body from various environmental hazards, such as exposure to harmful chemicals, ultraviolet radiation, and pathogenic organisms. Damage to the skin is a common phenomenon and a unique challenge, as a wound therapeutic is a complex process, which requires a considerable amount of time. Many wounds are unresponsive to existing treatments, thereby making these wounds a cumulative threat to public health and the economy [1]. Currently, drug resistance has been shown to be a common occurrence among many bacteria, including *Escherichia coli* and *Staphylococcus aureus* species (super-bugs) [2,3]. Thus, there is an urgent need to identify biogenic effective nanomaterials with broad-spectrum antibacterial properties to tackle the current problem of wound healing.

**Figure 1:**
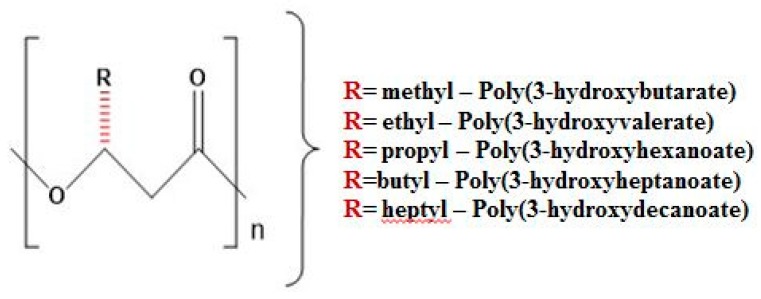
General Structure of Polyhydroxyalkanoate.

Many efforts have made for better wound management by making extensive use of biocompatible nanomaterials. The ideal biomaterial for wound dressing must possess properties such as biocompatibility, gaseous exchange, flexibility, biodegradability, and being a safeguard to prevent bacterial infection and remove excess exudates [4,5]. Some of the commonly used polymers in medicine, namely silicones, have been suspected to cause cancer and thus must be replaced by some biocompatible polymers [6]. Among various available biogenic polymers, polyhydroxyalkanoate (PHA) is a biopolymer produced by various bacterial cells under imbalanced growth conditions [7]. The general structure of PHA is depicted in Figure 1. PHA is a widely investigated biopolymer and is well-known for its excellent properties, namely its nontoxic nature, biocompatibility, and biodegradability [8,9,10]. The degradation product of poly 3-hydroxybutrate (P3HB) is a common metabolite in all higher living beings, which is an important aspect of PHA [11]. In recent years, poly 4-hydroxybutyrate (P4HB), poly 3-hydroxybutrate, and the copolymer poly 3-hydroxybutyrate-*co*-3-hydroxyhexanoate (PHBHHx) [12,13] have been widely used in the field of medicine to develop biologically significant devices, including sutures, cardiovascular scaffolds, orthopedic scaffolds, and adhesion barriers as well as guided tissue repair, nerve guide, tendon repair, and wound dressings [14,15,16,17,18,19]. The versatile structure of PHA can be modified simply through physical blending and chemical alteration to improve its efficacy for medicinal use. However, reinforcement of nanoparticles and nanocomposites feasibly enhances the resulting scaffold with multiple functionalities for efficient therapeutic and tissue engineering applications.

Graphene is a two-dimensional sheet of sp^2^-hybridized carbon atoms densely packed into a honeycomb lattice [20]. Graphene and its derivatives have received much attention for their excellent physicochemical properties, such as their good thermal and optical properties, high surface area to volume ratio, high strength, good elastic properties, ease of functionalization, and chemical inertness [21,22,23]. Due to these distinctive properties, graphene has often been used as a suitable matrix for designing biomedical devices with feasible substances, including polymers and nanoparticles. In the past decade, graphene has been widely investigated for its antibacterial, antiplatelet, anticancer, and biosensor activities [24,25,26]. Reduced graphene oxide (rGO) exerts its antibacterial property through the oxidizing agent glutathione that serves as a redox state mediator in bacteria and also disrupts the bacterial cell membrane upon direct contact [27]. Apart from antibacterial applications, graphene has been used in various applications in the fields of sensors, supercapacitors, energy storage devices, fuel cells, and high-strength materials [28,29,30]. Several important features of graphene make it a potential candidate for biomedical use. One of the most attractive features of graphene is its high surface area to volume ratio (2630 m^2^·g^−1^), which makes it a suitable candidate for nucleation of various nanoparticles. To improve the physicochemical properties of rGO, nanoparticles have been incorporated within its matrix by physical, chemical, and biological approaches. In recent years, rGO-based nanocomposites have been reported with metal nanoparticles, specifically with gold, silver, and platinum [31]. Nanoparticles or nanocomposites are now considered to be a feasible alternative to antibiotics and perhaps have a high potential to solve the emergence of bacterial multidrug resistance [32].

Among the noble metals, silver nanoparticles (Ag-NPs) have been extensively investigated due to their ease of synthesis and unique properties, including their antiseptic and antibacterial properties, their low cytotoxicity, and their high electrical and thermal conductivity [33,34]. Ag-NPs have found application in diverse fields, such as optical sensors, effective contrast agents, textile engineering, electronics, bactericidal agents, dental resin composites, coatings of medical devices, water filters, air sanitizer sprays, pillows, respirators, wet wipes, detergents, toothpastes, bone cements, wound dressings, and therapeutic agents. Sanitization through Ag-NPs was reported to be extremely toxic to bacterial cells depending on their size and distribution [35,36,37]. The mode of antibacterial action of silver nanoparticles is that it interacts with the sulfate group on the bacterial cell surface that blocks some enzymes responsible for energy metabolism and electrolyte transport. Thus, the oxidation stress damages the membrane, and the lack of enzyme suffocates and enforces bactericidal phenomena [38]. However, the size-related surface energy stimulates the agglomeration of Ag-NPs, which may reduce the efficacy of the antibacterial properties. To minimize Ag-NPs agglomeration, rGO with a high surface area is an excellent supporting matrix [39]. The synergistic antibacterial effect of rGO and Ag-NPs in a nanocomposite (hereafter denoted GAg) makes it an ideal antibacterial agent with dual killing effect [40,41,42]. Numerous physical and chemical protocols have been reported in the literature to synthesize graphene and silver nanoparticles. However, these methods are expensive or use toxic substances that make them incompatible with medical applications. The more suitable way to synthesize a biocompatible and less-toxic rGO and Ag-NPs composite is the use of green materials, such as a plant extract [39,43]. Nanomaterials synthesized using green techniques are less toxic, eco-friendly, and compatible with biomedical applications [44]. Recently, many techniques have been reported using plant and fungal extracts for the biosynthesis of a GAg nanocomposite [45]. In this investigation, a GAg nanocomposite is synthesized biogenically using cheaply available *Ganoderma lucidium* (G.L.) extract as a strong reducing agent [46]. The rich content of polysaccharide (glucans) in G.L. extract assists the reduction of graphene oxide (GO) and silver nitrate in a single reaction to produce a GAg nanocomposite. Electrospinning has gained lots of scientific consideration owing to its ability to produce biomedically attractive materials within nanoscale regime since the as-spun fibres mimic the nanoscale properties of the native extracellular matrix. Electrospun fibrous porous biomaterials have been investigated as promising tissue engineering, antibacterial, and wound dressing scaffolds [8,9,47].

In the present study, we intended to integrate the broad-spectrum antimicrobial activity of a GAg nanocomposite with the biocompatibility of P3HB-*co*-12 mol.% HHx (hereafter denoted PHA) through an electrospinning technique. The outstanding properties of PHA, comprising its biocompatible nature, enhanced biodegradability at a set time, and flexibility, are suitable features to support wound healing. Alongside this, it has been assumed that in a GAg nanocomposite, Ag-NPs loaded onto rGO may feasibly allow for stabilization and curb agglomeration of the nanoparticles. The narrow size distribution of Ag-NPs on the high surface area of rGO maximizes the direct contact of nanoparticles with bacterial cells, which improves the bactericidal activity. To the best of our knowledge, there is no report on the application of as-spun PHA/GAg nanocomposite mats for antibacterial studies. It is hypothesized that a PHA/GAg scaffold will exhibit dual bactericidal properties. The antimicrobial activities of the as-spun mats have been tested in triplicates among the batches of PHA/GAg scaffold against *E. coli* and *S. aureus*.

## 2. Materials and Methods

Graphite powder was received from Asbury Graphite Mill Inc., (Asbury, NJ, USA). Silver nitrate (AgNO_3_) and sodium hydroxide (NaOH) were obtained from Sigma-Aldrich, (St. Louis, MO, USA). Chloroform (CHCl_3_) and dimethylformamide (DMF) solvents of analytical grade were purchased from Sigma-Aldrich, Malaysia. *Ganoderma lucidium* powder was received from Ganofarm Sdn. Bhd., (Port Dickson, Malaysia). P3HB-*co*-12 mol.% HHx (350,000 Da) was provided by KANEKA corporation, (Tokyo, Japan). Ultra-pure deionised (DI) water was obtained from a Milli-Q plus system, EMD millipore, (Burlington, MA, USA). Electrospinning of scaffolds was carried out using an Esprayer ES-2000, Fuence Co. Ltd., (Tokyo, Japan). The stationary-phase bacterial strains. *S. aureus* (12,600 ATCC) and *E. coli* (8739 ATCC) were used for antibacterial studies. To prepare stationary-phase cultures, diluted cultures have been grown for 16 h of time prior to testing.

### 2.1. Preparation of Reducing Agent, G.L. Extract

**Figure 2:**
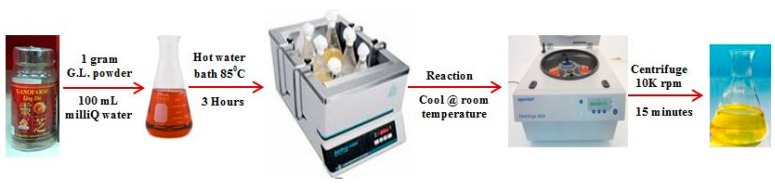
Schematic illustration of preparing *Ganoderma lucidium* extract.

In a 250 mL conical flask, 1 g of G.L. mushroom powder and 100 mL of Milli-Q grade water were added. This mixture was placed in a hot water bath for 3 h at 85 °C and then was cooled down at room temperature. The solution was transferred to 50 mL eppendrof tubes. To confiscate the suspended particles of G.L. mushroom, the reacted solution was centrifuged at 10,000 rpm for 15 min. The clear supernatant was collected carefully and used as a reducing agent directly or stored at 4 °C for further use. Figure 2 provides the pictorial illustration of the preparation of the G.L. extract.

### 2.2. Synthesis of GAg Nanocomposite

**Figure 3:**
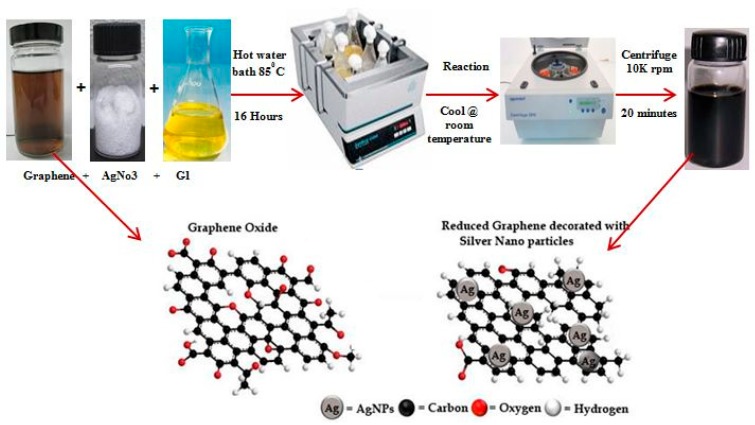
Green synthesis of the reduced graphene oxide (rGO) and silver nanoparticles (Ag-NPs) (GAg) nanocomposite.

GAg was synthesized using GO (graphite oxide) (prepared via a modified Hummers method using flake graphite), AgNO_3_, and G.L. mushroom extract. The pH of the GO solution (0.1 mg/mL) was adjusted to pH 7 by using NaOH. For synthesizing the GAg nanocomposite, to 25 mL of 0.1 mg/mL GO solution, 10 mM AgNO_3_ was added dropwise in a 250 mL conical flask containing 25 mL of G.L. extract. The mixture was then placed in a preheated rotary water bath maintained at the speed of 120 rpm at 85 °C for 16 h. The resulting solution was then allowed to cool at room temperature and was then transferred to 50 mL eppendrof tubes for centrifugation. The reaction mixture was centrifuged at 10,000 rpm for 20 min and the supernatant discarded. The resulting solid residue was again dispersed in DI water and washed through a centrifuge to remove all the residuals of the G.L. extract. This step was repeated five times in order to obtain a pure GAg nanocomposite. Since the G.L. extract is water soluble, it is easy to remove entirely from the reaction solution with simply a water-washing technique. To validate the reproducibility of the GAg using G.L. mushroom extract, all the experiments were repeated thrice under identical experimental conditions. Finally, the resultant GAg nanocomposite pallet was gently re-dispersed in Milli-Q water for further use. Figure 3 shows the schematic description of the GAg nanocomposite.

### 2.3. Fabrication of Electrospun Scaffold (PHA/GAg)

**Figure 4:**
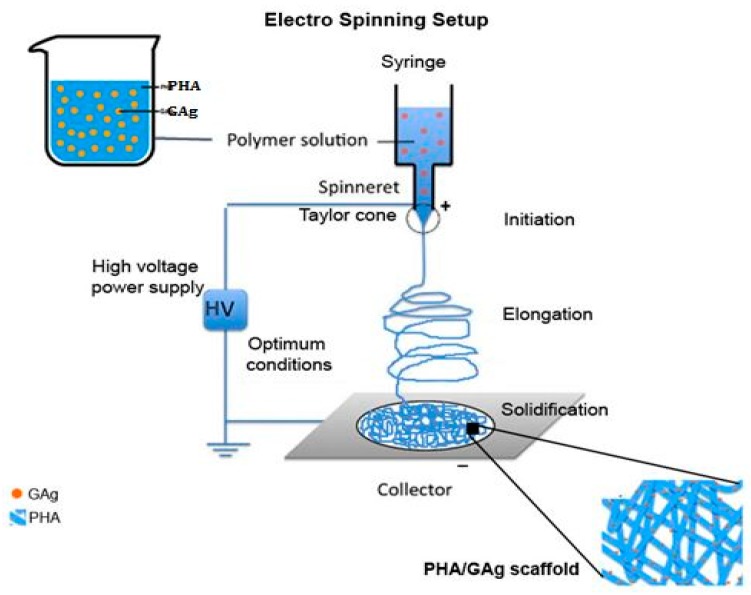
Schematic diagram of electro spinning PHA/GAg scaffold.

An electrospinning precursor solution was prepared using the organic solvents CHCl_3_ and DMF (8:2 ratio) [48] containing 5 mL of 3% (*w*/*v*) of P3HB-*co*-12 mol.% HHx and 15 μL of GAg. The precursor solution was subject to magnetic stirring for 48 h at room temperature followed by 2 h heat treatment at 55 °C to dissolve PHA completely. It was then sonicated in a water bath for 5 min prior to electrospinning to have an even distribution of GAg in the PHA solution. Similar steps were applied to make the precursor solution of rGO (reduced graphene oxide) and the control (PHA alone). An Esprayer ES-2000 (Fuence, Co. Ltd., Tokyo, Japan) was used to electrospin the nanocomposite mats. PHA/GAg mats (Figure 4) were produced at an applied voltage of 25 kV and an extrusion rate of 40 μL/min at the fixed distance of 20 cm from the needle tip to the copper plate collector. A total of 5 mL of PHA/GAg precursor solution was electrospun to obtain a scaffold of 4 cm × 4 cm. The obtained product of a PHA/GAg scaffold was then vaccum-dried for 48 h to eliminate any residuals of CHCL_3_ and DMF. Through the electrospinning procedure, the GAg nanocomposite has been eventually distributed and embedded on the as-spun fibres of PHA, which makes PHA/GAg an effective antibacterial scaffold. 

### 2.4. Antibacterial Culture Preparation

Three various batches of as-spun PHA/GAg, rGO, and a control mat with the disc size of 0.6 mm were tested against *S. aureus* (12,600 ATCC) and *E. coli* (8739 ATCC) as the model organisms by using a time kill test technique and the analysis was averaged out for all experiments. Diluted cultures were grown up to 6 h in 100 mL of nutrient broth prior to testing. Prepared precursor broth containing test organisms was transferred in equal amounts to sterile bijous bottles of size 15 mL. Moreover, discs of test scaffolds (PHA/GAg, PHA/rGO, and PHA) were exposed to UV for 30 min and then placed aseptically in precursor broth aliquots into the bottles. The aliquot in the control bottle treated with a PHA disc alone was a negative control. In the following analysis, gentamicin was used as a positive control for 100% kill. The bottles have been incubated at 37 °C in a shaker incubator at 150 rpm. The samples were collected at different time intervals at 0, 2, 4, 6, and 24 h and then serially diluted until the optimized working dilution was obtained. Finally, a 100 μL precursor broth sample treated with as-spun nanocomposite mats was aspirated and dispensed onto a freshly prepared nutrient agar plate with two duplicates each followed by the spread plate technique and incubated at 37 °C overnight to obtain the antibacterial activity of the as-spun PHA/GAg and PHA/rGO scaffolds.

## 3. Characterization

The surface structure and morphology of the PHA/GAg mats was studied using FESEM (Hitachi nano DUE’T NB5000, Schaumburg, IL, USA). The rGO was examined using the ultraviolet–visible (UV–Vis) spectrum at predetermined time intervals using a Lambda 35 Spectrophotometer (Perkin Elmer, Waltham, MA, USA). The particle size and stability were studied using a zetasizer Nano ZS (Malvern Instruments, Malvern, UK). Ultrasonication was employed by using a Cole Parmer dipstick sonicator (20 kHz ± 5 kHz) using a tapered microtip of 3 mm and an amplitude of 44% prior to zetasizer analysis. FTIR spectra of the PHA/GAg were obtained using a Spectrum RX1 (Perkin Elmer, Waltham, MA, USA) in the frequency range of 4000–400 cm^−1^. The thermogravimetric analysis (TGA) was performed by heating the samples from 25 to 1000 °C at the heating rate of 5 °C/min under a nitrogen flow (50 mL/min) using a TGA/differential scanning calorimetry 1 Stare System (Mettler Toledo Inc., Columbus, OH, USA). 

## 4. Results

### 4.1. FESEM and Elemental Analysis

**Figure 5:**
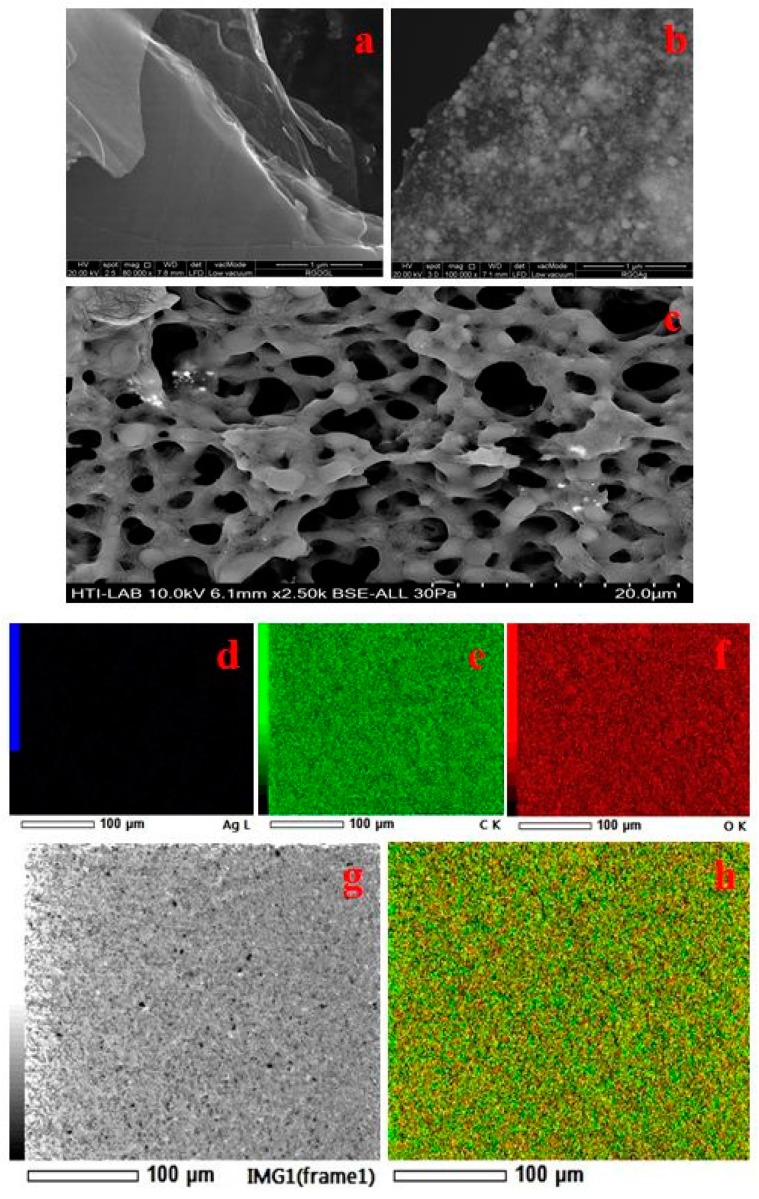
SEM images for the rGO (**a**), Gag (**b**), and PHA/GAg electrospun mats (**c**). Elemental mapping of a sample PHA/GAg scaffold is shown in figure (**d**–**h**) respectively.

The surface morphology of the synthesized material was comprehensively analysed by the FESEM technique. Figure 5 presents the FESEM micrographs of rGO, GAg, and PHA/GAg electrospun mats. As is evident from Figure 5a, rGO exhibits a transparent, rippled, and silk-like waves-and-wrinkles type of morphology. The ripples in micrograph of rGO represents the folded graphene sheets due to stacking. Deletion of strain on the C-C bond in the epoxy groups forms the wrinkles [49]. The obtained result is in good agreement with many recent studies depicting graphene sheets obtained from GO by various techniques, which possess curled morphologies consisting of thin, wrinkled, paper-like structures with fewer layers and having larger specific surface areas than GO [50]. The FESEM micrograph of Ag-NPs reveals the formation of spherical particles within nano-range as depicted in Figure 5b. The morphological analysis of the GAg nanocomposite decorated on PHA fibres is illustrated by Figure 5c. As is apparent from Figure 5c, there is a formation of a porous mat-like matrix with fibrous morphology indicating the formation of a fibrous PHA/GAg nanocomposite.

However, it is difficult to locate silver nanoparticles on the PHA/GAg nanocomposite since these particles are embedded deep within the polymer matrix and hard to visualise by FESEM analysis. Thus, elemental mapping appears to be the best technique to establish the uniform presence of Ag nanoparticles within the matrix of PHA polymer. Figure 5d–g and h represent the elemental mapping analysis of the PHA/GAg nanocomposite, which evidently reveals that Ag nanoparticles are homogeneously present within the matrix of the composite material along with carbon and oxygen.

### 4.2. UV-Vis Analysis

**Figure 6:**
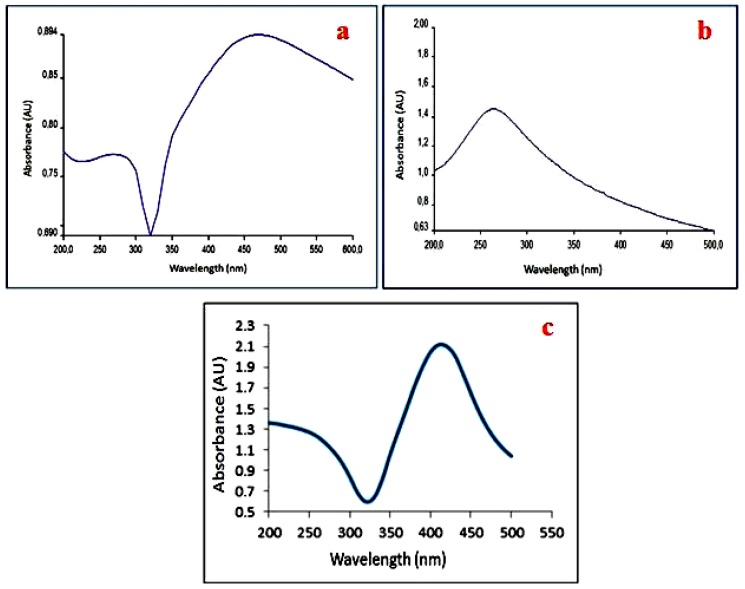
UV-vis spectra obtained after the green synthesis of Ag-NPs (**a**), rGO (**b**), and GAg (**c**).

Figure 6 displays the UV-vis absorbance spectrum for the Ag nanoparticles, rGO, and PHA/GAg, respectively. As is evident from Figure 6a, the UV-vis spectrum of Ag-NPs exhibits a distinct broad absorption peak at about 410 nm, which is the characteristic peak for Ag and indicates the successful formation of Ag-NPs [51]. On the other hand, the UV-vis spectrum of rGO (Figure 6b) demonstrates a broad band at 260 nm, which can be attributed to the conjugated sp^2^ carbon network in rGO and is a characteristic of rGO [52]. No significant plasmon peak appears at 300 nm in the spectrum of rGO, suggesting the removal of oxygen groups and thereby confirming the successful reduction of GO to rGO [53]. Moreover, the characteristic peak of GO appears at 230 nm, which is absent in the spectrum of rGO, thereby confirming the formation of rGO. As is apparent from Figure 6c, the UV-vis spectrum of GAg reveals the presence of the characteristic peaks for both Ag and rGO, thereby demonstrating the successful synthesis of Ag-nanoparticles-decorated rGO.

### 4.3. Particle Size Analysis

**Figure 7:**
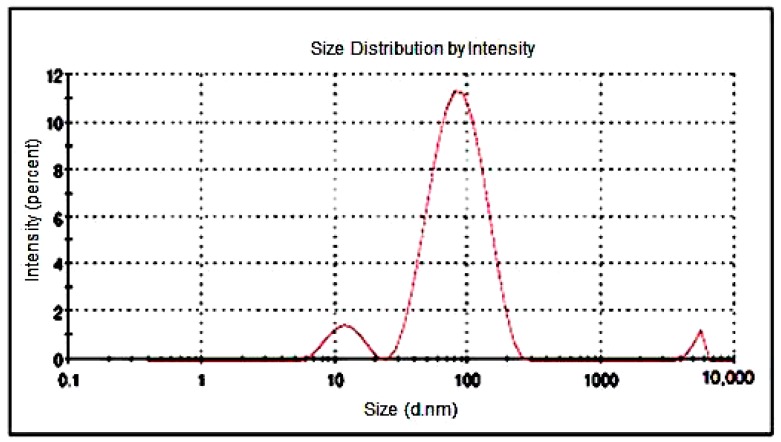
Dynamic light scatter of GAg nanocomposite.

Furthermore, the particle size distribution of GAg was determined by DLS (Dynamic Light Scattering) analysis, which is the most valuable and useful technique to assess the particle size and size distribution of any nanomaterial in solution [54]. The results of DLS demonstrated that the size distribution of Ag-NPs within GAg was found to be range from 10 to 80 nm in diameter. The z-average diameter noted for Ag-NPs has been found to be 66.52 nm (Figure 7). The DLS technique can be used for the study of rGO-based composites, but the dimensions of rGO sheets and GO-based nanocomposites determined by this technique do not represent the real particle size [55]. Though DLS is appropriate for spherical particles rather than planar sheets, such as rGO, it serves to indicate whether a uniformly sized dispersion of grapheme flakes has been produced. The particle size distribution in the case of GAg as detected by DLS exhibited the presence of particles around 30 to 120 nm that may be attributed to the agglomeration of Ag-NPs or rGO flakes. Moreover, the presence of a small peak on the left may be attributed to non-binded silver nanoparticles whereas another smaller peak on the right may be due to agglomeration of nanoparticles with r-GO.

### 4.4. FTIR Analysis

**Figure 8:**
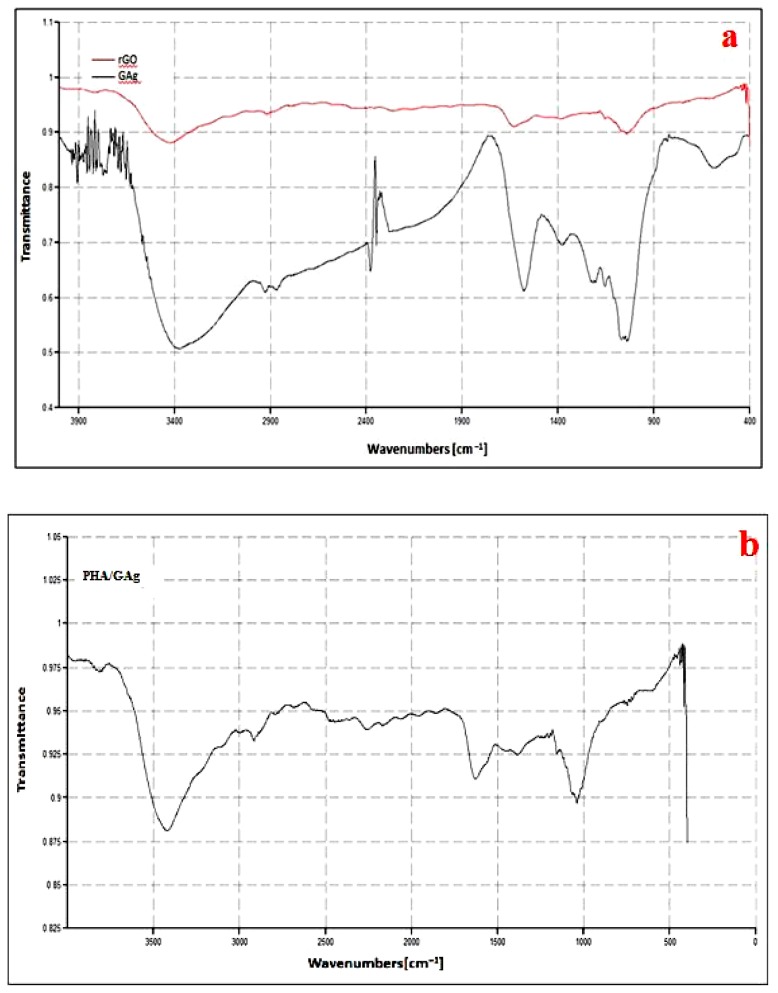
FTIR spectra of rGO and GAg (**a**) and PHA/GAg (**b**) nanocomposites.

The FTIR spectra of rGO nanoflakes and GAg are shown in Figure 8a and those of PHA-embedded GAg nanocomposites are shown in Figure 8b. The IR bands appearing at approximately 3414, 1725, 1626, 1245, and 1060 cm^−1^ of rGO are assigned to -OH stretching vibrations, C=O stretching of Carboxylic groups, -OH deformations of the C-OH groups, epoxy symmetrical ring deformation vibrations, and C-O stretching vibrations, respectively [56]. The peaks at 1060 cm^−1^ (C-O stretching vibrations), 1245 cm^−1^ (C-OH stretching vibration), and 1725 cm^−1^ (C=O stretching vibration) had become less intense, which suggests the reduction of graphene. The peaks between 2924 cm^−1^ and 3400 cm^−1^ of silver were assigned to the stretching vibrations of primary and secondary amines, while their corresponding bending vibrations were seen at 1383 cm^−1^ and 1636 cm^−1^, respectively [57]. The wave number positioned around 1700 cm^−1^ was attributed to the stretching vibration of the C=O group (ester carbonyl) in the PHA. Associated bands of the C-O-C groups appeared in the spectral region of 1150 to 1300 cm^−1^. P3HB-*co*-HHx had the strongest methylene -C-H- vibrations at around 2933 cm^−1^. In addition, the wavenumber at 3436 cm^−1^ is assigned to the OH vibration in the carboxyl group of P(3HB-*co*-3HHx) [58,59]. The overall observation of the FTIR band confirms the successful formation of a PHA/GAg composite mat.

### 4.5. Thermal Analysis

**Figure 9:**
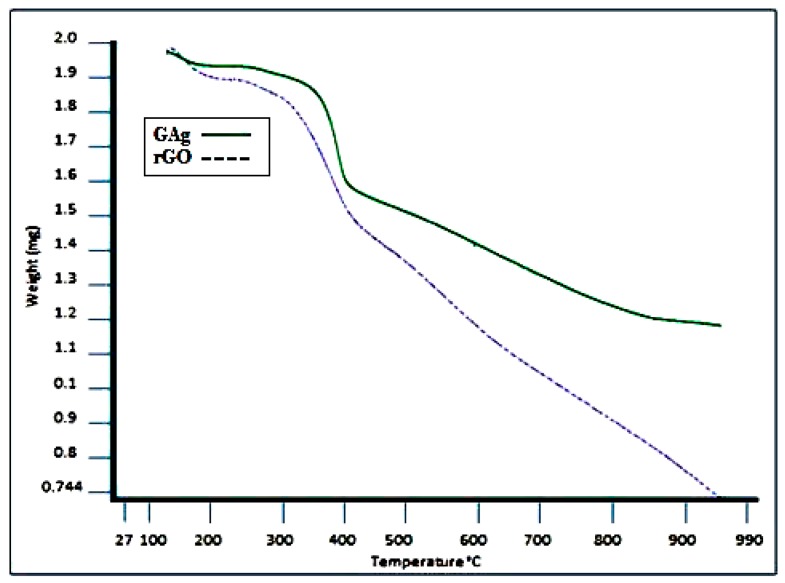
Thermogravimetric (TGA) spectra of rGO and GAg nanocomposites.

The thermal stability of GAg and rGO was analyzed using TGA. Figure 9 exhibits the TGA curves of rGO and the GAg nanocomposite under a nitrogen atmosphere. The decomposition of rGO and GAg begins with moderate weight loss at around 100 to 250 °C that is probably due to the removal of water molecules, the oxygen functional group, and residues. The maximum weight loss was observed from 250 to 400 °C for both rGO (38%) and GAg (28%), which may be due to the loss of the left over oxygen functional group and bulk pyrolysis of the carbon skeleton. The TGA curves of rGO and GAg exhibit a slight mass loss at the temperature of around 600 °C, which suggests that the enhancement of thermal stability is achieved after the oxygen-containing functional groups were removed during reduction [60,61]. The TGA results of GAg showed better thermal stability as compared to rGO alone. GAg has less oxygen groups and contains Ag-NPs that can affect the thermal stability of graphene, suggesting strong interaction between rGO and Ag.

### 4.6. Antibacterial Analysis

The antibacterial activity of the as-spun nanocomposite was obtained against model organisms *E. coli* and *S. aureus* through the time kill method. Sterile bottles containing 0.6 mm discs of PHA, PHA/rGO, and the PHA/GAg nanocomposite were aliquoted with 5 mL of culture broth of each test organism separately. Gentamicin was used as a positive control, which is a broad spectrum antibiotic with effective bactericidal activities. Its mode of action includes inhibition of bacterial protein synthesis by binding to 30S ribosomes inside the bacteria cell. The aliquot in the control bottle was treated with a PHA disc alone as a negative control. All bottles were agitated at 150 rpm in a shaker incubator at the temperature of 37 °C. After the set time intervals, 100 µL culture broths were aspirated from all the samples and dispensed onto freshly prepared nutrient agar plates with three replicate of each sample followed by the spread plate technique under sterile conditions and incubation at 37 °C overnight (14 h).

**Figure 10:**
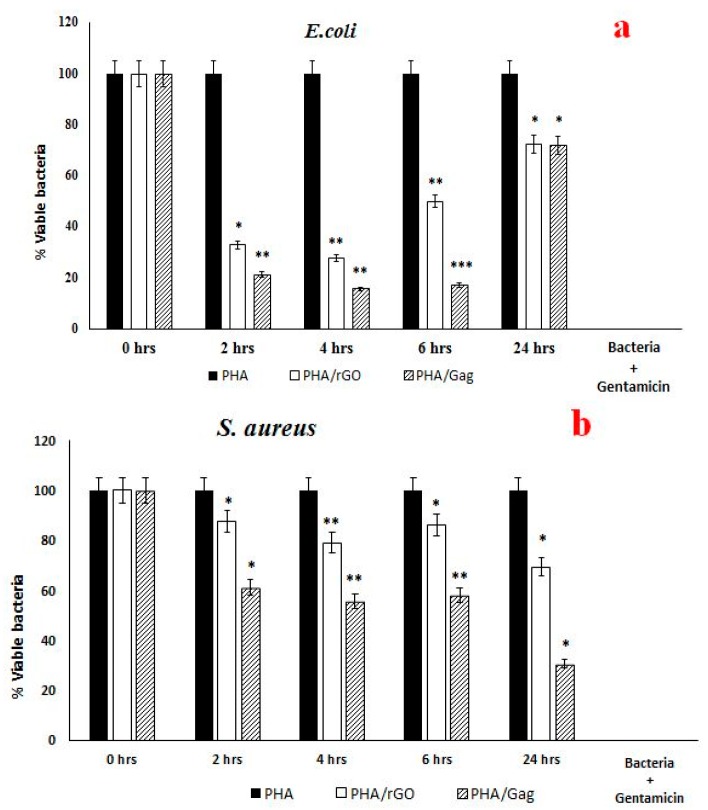
Bactericidal activity of PHA, PHA/rGO, and PHA/GAg against test organisms. PHA/rGO and PHA/GAg showed significant (*p* value <0.05) bactericidal effects towards *Escherichia coli* (**a**), and *Staphylococcus aureus* (**b**). *p* values were determined using a two-sample *t*-test distribution, (*) is <0.05; (**) is <0.01; and (***) is <0.001. The result presented was the average of three different batches of as-spun scaffolds of each nanocomposites (PHA, PHA/rGO and PHA/GAg.

The antibacterial activities of the as-spun nanocomposites of three various batch scaffolds, have been evaluated at different time intervals. The average results presented here, is (In) the first 2 and 4 h time intervals, the bactericidal activity of the tested samples against *E. coli* and *S. aureus* was highly significant with respect to the 6- and 24-h samples. Moreover, up to 24 h, PHA/rGO and the PHA/GAg nanocomposite have shown significant reduction of bacterial cells in comparison with the negative control. Significant colony forming unit (CFU) pls define percent inhibition was shown by *E. coli* inoculated with different test nanocomposites. The *p* value (*p* < 0.05) was obtained by using a two-sample *t*-test distribution: (*) is <0.05; (**) is <0.01; and (***) is <0.001 (Figure 10a). Similarly, *S. aureus* has also shown significant CFU percent inhibition towards the tested nanocomposite (*p* < 0.05 using a two-sample *t*-test distribution: (*) is <0.05; (**) is <0.01; and (***) is <0.001) (Figure 10b). These statistical analyses are further supported in Table 1.

Herein, the 4-h bactericidal activity of PHA/GAg against *E. coli* has shown (Figure S1) highly significant activity compared with the negative control and less significant activity as compared to PHA/rGO. *S. aureus* treated with PHA/GAg demonstrated significant bactericidal activity (Figure S2) in comparison with PHA/rGO and the negative control. Significant bacterial inhibition obtained up to 4-h of incubation and thereafter a gradual decrease was observed until 24 h in the test nanocomposites compared to the control. Biogenically as-spun nanocomposites have shown less bactericidal activity with respect to commercial gentamicin.

## 5. Conclusions

In conclusion, PHA/rGO and PHA/GAg nanocomposites have been successfully fabricated. The resultant scaffolds were tested for their antibacterial properties against test organisms. Significant bactericidal activity was observed in the first 2 and 4 h time intervals compared to 6 and 24 h. It is hypothesized that silver ions have been actively released into the inoculum for the first 4-h time interval followed by gradual release until 24 h. *E. coli* has shown more sensitivity towards the tested nanocomposites as compared to *S. aureus*. PHA/GAg has shown significant bactericidal activity compared to PHA/rGO and the negative control. Antibacterial activities were recorded up to 24 h of incubation, which is an important factor for an effective wound dressing. Therefore, the as-spun PHA/GAg nanocomposite may feasibly be efficient in the treatment of chronic wounds and sanitizing applications.

## Figures and Tables

**Table 1:** Representation of PHA, PHA/rGO, and PHA/GAg scaffolds’ antibacterial activity against *E. coli* and *S. aureus.*

Test Samples	Antibacterial Activity against *E. coli*	Antibacterial Activity against *S. aureus*
PHA	−	−
PHA/rGO	+	+
PHA/GAg	+	+
Gentamicin	+	+

## Supplementary Materials

The following are available online at http://www.mdpi.com/1996-1944/11/9/1673/s1, Figure S1, At the time interval of 4 h, the bactericidal activity of PHA, PHA/rGO, and PHA/GAg against *E. coli* was evaluated. Significant decrease observed in the CFU, which demonstrates the bactericidal activity of PHA/rGO and PHA/GAg compared to PHA alone. PHA/GAg is considered very effective with its dual mode of action (reduced graphene and silver nanoparticles) towards *E. coli* and is highly significant. Positive control data has not been presented here. Figure S2, The antibacterial activity after the 4-h time interval was evaluated for PHA, PHA/rGO, and PHA/GAg against *S. aureus*. A significant decrease was observed in the CFU, which demonstrates the bactericidal activity of PHA/rGO and PHA/GAg compared to PHA alone. PHA/rGO and PHA/GAg have shown less reduction compared to *E. coli*. Positive control data has not been presented here.
