# Supplementary material for: Fabrication and Characterization of an Electrospun PHA/Graphene Silver Nanocomposite Scaffold for Antibacterial Applications
Source: Materials (Basel). 2018 Sep 10;11(9):1673. doi: 10.3390/ma11091673 (PMC6163631; doi:10.3390/ma11091673)
Supplement: Supplementary file 1 [file materials-11-01673-s001.pdf]

Supporting Information

Journal: Materials
Title: **"Fabrication and Characterization of Electrospun PHA/Graphene Silver Nano-Composite Scaffold for Antibacterial Applications - Supplementary material"**

Authors: Abdul Mukheem, Kasturi Muthoosamy, Sivakumar Manickam, Kumar Sudesh, Syed Shahabuddin, Saidur Rahman, N. Akbar, Nanthini Sridewi

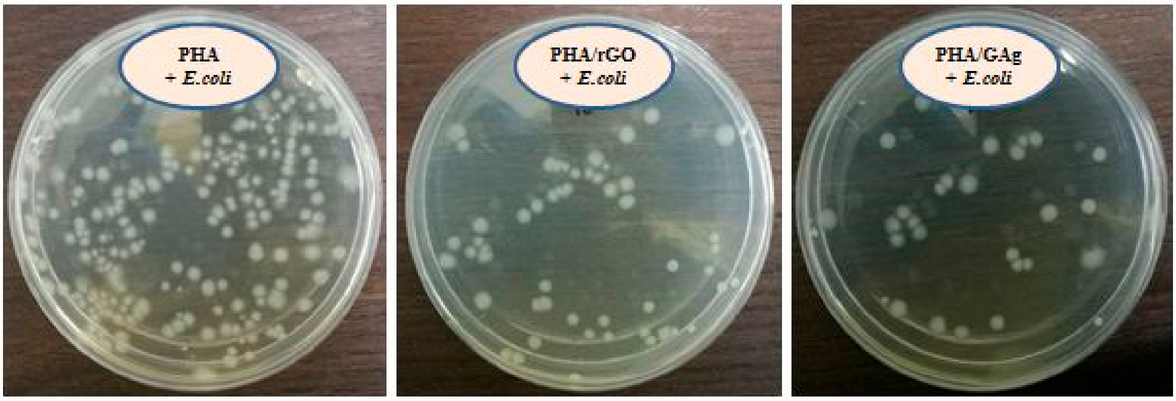

Figure S1 At the time intravel of 4 h bactericidal activity of PHA, PHA/rGO and PHA/GAg against E.coli was evaluated. Significant decrease is observed in the CFU which demonstrates the bactericidal activity of PHA/rGO and PHA/GAg compare to PHA alone. PHA/GAg is consider very effective with its dual mode of action (reduced graphene and silver nanoparticles) towards the E.coli and is highly significant. Positive control data has not presented here

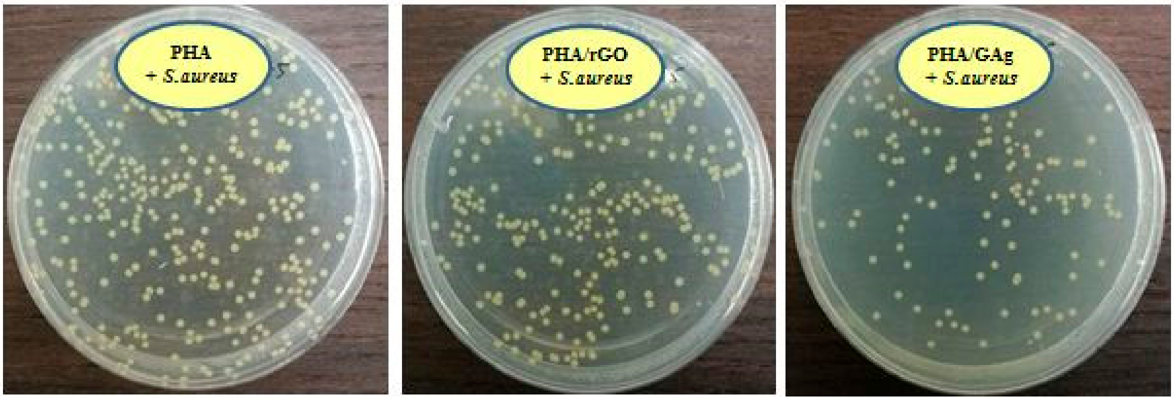

Figure S2 The antibacterial activity after 4 h time intraval, was evaluated for PHA, PHA/rGO and PHA/GAg against S.aureus. Significant decrease is observed in the CFU which demonstrates the bactericidal activity of PHA/rGO and PHA/GAg compare to PHA alone. PHA/rGO and PHA/GAg has shown less reduction compare to E.coli. Positive control data has not presented here
